# Effects on stress reduction of a modified mindfulness-based cognitive therapy for family caregivers of those with dementia: study protocol for a randomized controlled trial

**DOI:** 10.1186/s13063-019-3432-2

**Published:** 2019-05-29

**Authors:** Patrick Pui Kin Kor, Justina Yat-Wa Liu, Wai Tong Chien

**Affiliations:** 1 Centre for Gerontological Nursing, School of Nursing, The Hong Kong Polytechnic University, Hong Kong, China; 2 The Nethersole School of Nursing, The Chinese University of Hong Kong, Hong Kong, China

**Keywords:** Mindfulness, Dementia, Caregivers, Stress, Family, Older people, Nursing, Community, Quality of life

## Abstract

**Background:**

Caring for people with dementia (PWD) is stressful and poses many life challenges for the family caregivers. Interventions targeting the stress and psychological well-being of the caregivers have been proposed but the sustainable effects and efficacies of these interventions vary considerably. Mindfulness-based cognitive therapy (MBCT) has been shown to be effective at reducing stress in several populations. However, limited research on the effects of MBCT in family caregivers of PWD has been conducted. This study protocol aims to examine the effects on stress reduction of a modified MBCT for family caregivers of PWD.

**Methods:**

A prospective, single-blind, parallel-group, randomized controlled trial will be adopted. A convenience sample of 100 community-dwelling family caregivers of PWD will be randomized to either the modified MBCT or the control groups. The modified MBCT group will receive a 10-week, seven-session, group-based modified MBCT whereas the control group will receive a social interaction and routine education (SIRE) on dementia care program at a frequency and timing similar to those in the intervention group. The primary outcomes (stress) and secondary outcomes (depression, anxiety, burden, health-related quality of life, and the behavioral and psychological symptoms of the care recipient) will be measured immediately post-intervention (T1) and at 6-month follow-up (T2), which will be compared with the baseline (T0).

**Discussion:**

Reducing the stress of caregiving can promote the well-being of the family caregivers and maintain their sustainability in providing daily care for their family members with dementia. MBCT is found to be effective for stress reduction in other populations, and the results of this study are able to provide us with evidence for using MBCT as a standard supportive intervention for the family caregivers of PWD.

**Trial registration:**

ClinicalTrials.gov, NCT03354819. Registered on 28 November 2017.

**Electronic supplementary material:**

The online version of this article (10.1186/s13063-019-3432-2) contains supplementary material, which is available to authorized users.

## Background

Dementia is a common neurodegenerative disease in older people causing gradual cognitive decline and a series of behavioral and psychological symptoms of dementia (BPSD). As the disease progresses, the self-care ability of people with dementia (PWD) will gradually be lost. Family caregivers play an important role in caring for PWD, and this challenging role usually results in chronic stress and negative emotions [1]. Nowadays, there are different types of nonpharmacological interventions designed for the caregivers. However, the existing evidence supporting the current interventions in stress reduction for family caregivers of PWD is still weak with a small effect size (Cohen’s *d* = 0.18–0.27) [2]. Some studies found that support groups and respite care cannot significantly reduce the stress level of caregivers, although they can provide caregivers with skills, knowledge, and transient support on caring for PWD [3, 4].

Stress is a two-way process that involves the presence of the stressors in the environment and the response and appraisal of an individual subjected to those stressors [5]. Most of the current interventions such as respite care adopt a problem-solving approach and aim to manipulate the stressors of the family caregiver of PWD. However, caregiving stress is chronic and most of it is related to the cognitive decline of the care recipients which is difficult to modify. Thus, an emotion-focused approach is usually adopted for managing stress in the caregivers of PWD [6].

In the past few decades, mindfulness-based intervention (MBI) has been found to be effective at improving a few of the main psychological symptoms, including anxiety, depression, psychotic symptoms, and stress [7–12]. The most commonly used MBI is the Mindfulness-Based Stress Reduction (MBSR) program [13] and mindfulness-based cognitive therapy (MBCT) [14]. The MBSR was developed by Jon Kabat-Zinn in 1970 at the Stress Reduction Clinic at the University of Massachusetts Medical School and was primarily designed for patients with chronic pain. It is a group-based and multi-component intervention involving a wide range of activities, including sitting meditation, mindful walking, mindful eating, body scanning, group sharing, home practice, and psychoeducation on stress and psychological health. Based on the MBSR, Segal et al. [14] developed the MBCT to prevent a lapse into depression in people with a major depressive disorder. Similar to the MBSR, the program consists of eight 2-h weekly sessions with a 1-day retreat. MBCT adopted the principles of MBSR and integrated cognitive-behavioral techniques that explore more on negative thinking and its impacts on the well-being of participants [14]. Compared with MBSR, MBCT involves imagining particular scenarios and asks the participants to notice what thoughts appear. At the same time, the participants are taught to focus less on reacting to incoming stimuli (stressors) and instead on accepting and observing the stimuli without judgment.

A systematic review was conducted by us which included five experimental studies to investigate the effect of MBI (including MBCT and MBSR) on stress reduction in the family caregivers of PWD [15]. The result showed that the stress levels dropped significantly after an 8-week MBI in the family caregivers of PWD with a moderate aggregated effect size of 0.57 (95% confidence interval (CI) 0.23–0.92, overall effect *Z* = 3.25; *p* = 0.001). While an immediate effect of MBI was seen, the long-term effect (i.e., 6 months after the intervention) was unclear. Furthermore, a limited number of clinical trials and several limitations (such as poor study design, small sample size, lack of information on the adherence rate when practicing mindfulness, and a high attrition rate) were also identified in this review. This signifies that more studies are still required to examine the effects of MBI for family caregivers of PWD.

MBI is a relatively new intervention for stress management in the caregivers of PWD. Therefore, a feasibility study including 53 family caregivers of PWD was conducted between 2016 and 2017 by us to investigate the feasibility of using MBI in family caregivers of PWD, and also to determine which therapeutic modality in mindfulness (MBSR or MBCT) is more effective in reducing stress in the caregivers [16]. The results showed that the family caregivers could master the mindfulness skill after the mindfulness sessions with a low attrition rate of 3.8%. Significant improvement was found for all psychological outcomes, including stress, depression, and burden, in both MBI groups. Compared with MBSR, the MBCT participants had significantly greater improvements in perceived stress (mean difference = 1.74, standard error = 0.78). Based on the suggestions from the focus group interview in the feasibility study, the MBCT protocol was further modified for this main study.

## Methods

### Study design

**Fig. 1:**
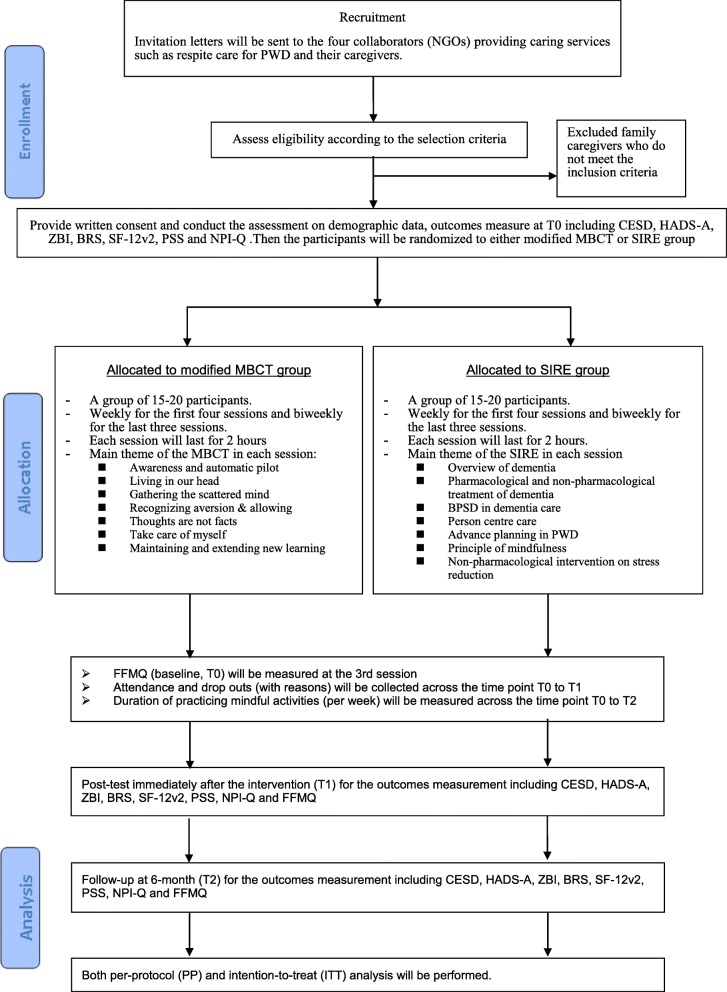
Consolidated Standards of Reporting Trials (CONSORT) flow diagram. BPSD Behavioral and psychological symptoms of dementia, BRS Brief Resilience Scale, CESDS Center for Epidemiologic Studies Depression Scale, FFMQ Five Facet Mindfulness Questionnaire, HADS-A Hospital Anxiety and Depression Scale-Anxiety, MBCT mindfulness-based cognitive therapy, NGO nongovernmental organization, NPI-Q Neuropsychiatric Inventory Questionnaire, PSS Perceived Stress Scale, PWD people with dementia, SF-12v2 Short-form 12-item health survey version 2, SIRE Social interaction and routine education, ZBI Zarit Burden Scale

**Fig. 2:**
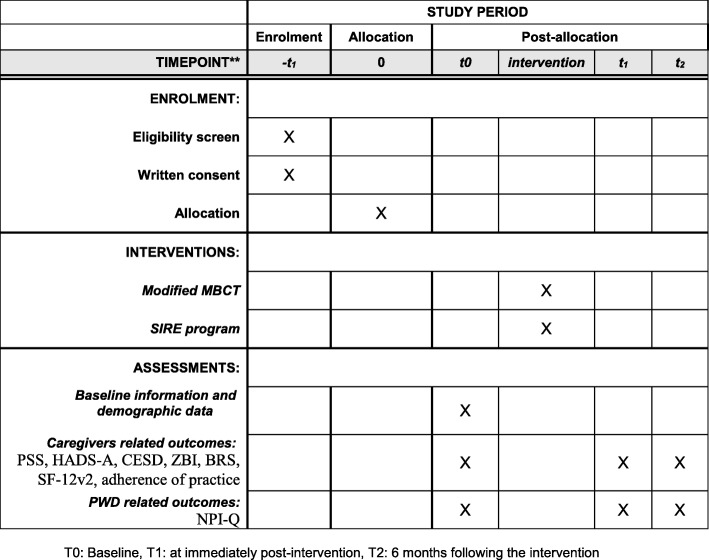
Standard Protocol Items: Recommendations for Interventional Trials (SPIRIT) figure. BRS Brief Resilience Scale, CESDS Center for Epidemiologic Studies Depression Scale, HADS-A Hospital Anxiety and Depression Scale-Anxiety, MBCT mindfulness-based cognitive therapy, NPI-Q Neuropsychiatric Inventory Questionnaire, PSS Perceived Stress Scale, PWD people with dementia, SF-12v2 Short-form 12-item health survey version 2, SIRE Social interaction and routine education, ZBI Zarit Burden Scale

This is a prospective, single-blind, parallel-group, randomized controlled trial (see Fig. 1 for the Consolidated Standards of Reporting Trials (CONSORT) flow diagram, Fig. 2 for the Standard Protocol Items: Recommendations for Interventional Trials (SPIRIT) figure, and Additional file 1 for the SPIRIT checklist).

### Objective

This study aims to investigate the effects of a modified MBCT on stress reduction in the family caregivers of PWD.

### Hypotheses of the study

Participants in the intervention group will have better improvement than the control group after the seven-session, 10-week modified MBCT intervention immediately after the intervention (T1) and/or at 6-month follow-up (T2) in terms of the following hypotheses: 1) significantly greater reduction of stress (primary outcome); and 2) significantly greater improvement in the secondary outcomes, namely depression, anxiety, resilience, burden, health-related quality of life, and the behavioral and psychological symptoms of the care recipient.

In addition, there are two hypotheses to facilitate the explanation of the positive changes in the modified MBCT group: 1) there will be significant positive correlations between the levels of mindfulness of the caregivers (total score of five facets of mindfulness) and the improvement in their stress, depression, anxiety, burden, resilience, health-related quality of life, and/or behavioral and psychological symptoms of the care recipient; and 2) increased use of all facets or individual facets of mindfulness skills of family caregivers will be significantly and positively correlated with the improvements in their psychological outcomes (stress, depression, anxiety, burden, resilience, and/or health-related quality of life).

### Study setting

The subjects will be recruited from three nongovernmental organizations (with collaborations before) in Hong Kong providing caring services such as respite care and cognitive training for PWD and their caregivers. The centers are located in the three largest districts in Hong Kong, namely Kowloon, Hong Kong Island, and the New Territories, serving more than 10,000 older people and their caregivers.

### Sample selection criteria

The target population is the community-dwelling family caregivers of PWD. Inclusion criteria are as follows: 1) those aged 18 years or older; 2) blood or by-marriage relatives (e.g., spouses, siblings, children, and grandchildren) of a person who has been clinically diagnosed with dementia, regardless of its types, with these relatives taking on the caring responsibilities ranging from physical aids to emotional support in the form of transportation, financial assistance, personal hygiene, and decision-making; 3) providing most of the daily care and support for PWD (daily contact for at least 4 h); and 4) able to speak Cantonese (for understanding all the teaching materials and instructions).

Caregivers will be excluded if they: 1) are practicing or recently learned meditation, mindfulness activities, and/or relaxation training in the past 6 months; 2) are diagnosed with a mental disorder such as bipolar disorder, schizophrenia, dementia, or depression in an acute stage; 3) are taking anticonvulsants or any kind of psychotropic drugs starting within the past 4 weeks which could be regulated or adjusted during the intervention period and thus the mental state might not be stable during the study; and/or 4) are identified with self-reported suicidal thought or drug abuse in the past 6 months.

The family caregivers receiving different types of support (other than MBI) such as domestic helpers, respite care, old age placement, old age allowance, meal delivery, or other financial support from the government will not be excluded, but the information will be reflected in the demographic data.

### Sample size

The sample size estimation was based on the primary outcome (stress). In the previous systematic review, the effect size of the MBCT for stress reduction was found to have Cohen’s *d* of 0.57 immediately after the intervention [15]. However, the intervention protocol is further modified and its duration is shortened in this proposed study. A conservative effect size Cohen’s *d* of 0.4 was adopted to detect the mean difference in stress reduction between the intervention and control groups. Based on the power analysis using the software G*Power 3.1 on repeated-measures analysis of variance (ANOVA), a sample size of 84 participants will be needed to achieve 80% power at a two-sided 5% significance level. According to the results of the feasibility study in Hong Kong, an attrition rate of 4% was found [16]. By adopting a more conservative estimation of the attrition rate of about 10%, the estimated sample size is at least 100 for this proposed study, 50 per group.

### Recruitment procedure

A convenience sampling method will be used to recruit participants. An invitation email will be sent to the three nongovernmental organizations providing elderly care services for PWD and their caregivers. If there are more than enough participants recruited from different centers, an equal proportion of participants will be randomly selected from different centers to maximize the diversity of the caregivers living in different districts.

### Randomization and blinding

The eligible participants will be randomized into the two groups (modified MBCT or social interaction and routine education (SIRE)) following an allocation concealment mechanism. To ensure equal sample sizes (1:1) in the modified MBCT and SIRE, the allocation of participants will be performed using a block randomization method and computer-generated numbers by an independent research assistant who will not be involved in the recruitment, assessment, or delivery of the intervention. The procedure and treatment allocation will be blinded to the investigators and outcomes assessors. The interventionists will be blinded to all the assessment results. The subjects will be told about their group assignment during the first session of the intervention via a sealed envelope.

### Interventions

#### The MBCT group

A group-based, 10-week, seven-session modified MBCT will be adopted in the intervention group (Table 1). Compared with the original MBCT protocol [14], the modified MBCT protocol in this pilot study has the following changes:

1. Integrating the contents of the third and fourth sessions into one session and abridging the full-day retreat (rationale: to promote the feasibility of complying with the intervention required for the caregivers). A study with a similar number of sessions of MBI demonstrated a moderate to large effect size on psychological outcomes in patients with cancer, which supports that the intervention of the present program may be sufficient [17].
2. Biweekly session for the last three sessions (rationale: facilitate the caregivers to apply mindfulness in daily lives and promote continuous home practice).
3. Telephone follow-up for the last three sessions (rationale: to monitor the progress of practicing mindfulness and answer simple questions or technical problems such as playing the CD).
4. Content about relapse prevention for a major depressive disorder, such as territory of depression in session 4 and relapse signature in session 5 in the original MBCT, will be replaced by psychoeducation about family caregiving stress and mindful communication (rationale: since MBCT was originally designed for people with recurrent depression, some content on relapse prevention for a major depressive disorder, such as territory of depression in session 4 and relapse signature in session 5, may not be applicable to caregivers of PWD). From the literature review, the family caregivers and the PWD usually have a conflict during their communication [18]. Mindful communication is an activity developed from the MBSR program and helps the participants to be mindful in the interactions with others including their family members. Several studies have adopted it for family caregivers of people with different diseases to improve their communication [19, 20].

**Table 1:** Modified mindfulness-based cognitive therapy (MBCT) program

Session	Main theme	Content	Home practice
1	Awareness and automatic pilot	➢ Establish the orientation of the class ➢ The Raisin exercise (eating meditation) ➢ 13-min body scan ➢ Feedback and discussion of exercise	➢ 45-min body scan for 6 out of 7 days ➢ Mindfulness of a routine practice
2	Living in our head	➢ Practice review ➢ Thoughts and feelings exercises ➢ 45-min body scan ➢ Brief mindfulness of breathing	➢ 45-min body scan for 6 out of 7 days ➢ 10 min of mindfulness of breath for 6 out of 7 days ➢ Pleasant experience calendar (one example daily) ➢ Mindfulness of a routine practice
3	Gathering the scattered mind	➢ Practice review ➢ Focus on unpleasant experiences exercises ➢ Seeing and hearing practice ➢ Sitting meditation (awareness of breath and body and responding to painful sensations) ➢ 3-min breathing space ➢ Mindful stretching and breath meditation ➢ Mindful movement	➢ Stretching and breathing meditation on days 1, 3, and 5 ➢ 40-min mindful movement on days 2, 4, and 6 ➢ Unpleasant experience calendar (a different experience each day) ➢ 3-min breathing space, three times daily
4	Recognizing aversion and allowing	➢ Practice review ➢ Seeing and hearing practice ➢ Mindful communication ➢ 3-min breathing space ➢ Mindful walking ➢ Sitting meditation (awareness of breath, body, and sounds, then how we relate to our experience through the reaction)	➢ Sitting meditation, 6 out of 7 days ➢ 3-min breathing space (three times a day) ➢ 3-min breathing space—responsive (whenever notice unpleasant feelings)
5	Thoughts are not facts	➢ Practice review ➢ Sitting meditation ➢ Mood, thoughts and alternative viewpoints exercise ➢ 3-min breathing space (responsive) ➢ Psychoeducation in family caregiving stress	➢ Select a guided meditation to practice at least 40 min per day ➢ 3-min breathing space (three times a day) ➢ 3-min breathing space—added instructions (whenever notice unpleasant feelings)
6	Take care of myself	➢ Practice review ➢ Sitting meditation with breath, body, sounds ➢ Activity and mood exercise ➢ Identifying habitual emotional reactions to difficulties ➢ 3-min breathing space (responsive)	➢ 3-min breathing space (three times a day) ➢ 3-min breathing space—added instructions (whenever notice unpleasant feelings) ➢ Select from all different forms of practice and apply them on a regular basis ➢ Develop action to be used in the face of lowered moods
7	Maintaining and extending new learning	➢ Practice review ➢ 45-min body scan ➢ Looking forward exercise ➢ Preparing for the future exercise	

The modified MBCT protocol was reviewed by an expert panel including mindfulness interventionists, nurses experienced in dementia care, and clinical psychologists to validate the contents of the protocol. Consensus among the experts on the contents of the MBCT was achieved through discussion of their suggestions. The protocol was also tested in the prior feasibility study and further modified for this main study.

#### Group size

Each group will have 15–20 participants to maintain sufficient group interactions between the MBCT instructor and all of the participants. A previous study indicated that the group effect accounted for 7% of the variability in the psychological outcomes (measured by the General Symptom Index) and that the group effect can be regarded as a booster in MBCT to improve the psychological outcomes [21]. In previous clinical trials on MBCT for people with recurrent depression, the group size was similar and varied from 12 to 14 [22–24]. The target population of our study is family caregivers without major depression. From the focus group of our prior feasibility study, most of the participants were satisfied with a group size of around 18 and indicated that they could discuss and share different caring experiences with their group colleagues [16]. There was also a significant change in mindfulness level immediately after the MBCT in the feasibility study, which indicated that most of the participants could master the skills of the mindfulness activities. Moreover, a smaller mindfulness group of 8 to 10 participants would not be cost-effective enough to accommodate or manage an increasing number of caregivers of PWD. In view of the previous literature and findings from the feasibility study, 15 to 20 participants were adopted as our group size.

#### Duration of sessions

The intervention will contain seven sessions and each session will last for about 2 h. A study with a similar number of MBI sessions demonstrated a moderate to large effect size on psychological outcomes in patients with cancer, which supports that the intervention of the present program may be sufficient [17]. The design of the original MBCT contained eight sessions with a 1-day retreat. Since family caregivers of PWD take up several roles and tasks in their daily life, most of the previous studies eliminated the retreat session to increase the adherence [20, 25, 26]. In our prior feasibility study, a number of the caregivers indicated that it was difficult to find time to join the whole-day retreat. Therefore, we eliminated the retreat session and combined sessions three and four in our feasibility study to shorten the duration. Eventually, the entire program contained seven sessions. Even though the length of each session was shortened in the feasibility study, a significant increase in the mindfulness level measured by the Five Facet Mindfulness Questionnaire (FFMQ) was identified, together with significant improvements in the psychological outcomes of the participants, immediately after the MBCT program. FFMQ is one of the most comprehensive and frequently used measures assessing individual differences in mindfulness skills and its psychometric property is described below. Therefore, we have adopted the same duration for the sessions here as in the feasibility study [16].

#### Frequency

The intervention will be set as weekly for the first four sessions and bi-weekly for the last three sessions. Since the participants were taught about the major components and skills of mindfulness in the first four sessions, the family caregivers could apply and practice the skills in their caregiving tasks. The caregivers return 2 weeks after the first four sessions to continue the bi-weekly sessions and they can then share more about the difficulties in applying mindfulness skills over the past 2 weeks. Compared with the original design of the MBCT protocol, the caregivers have more chances to practice in their daily lives which may facilitate the caregivers to develop a habit of daily mindfulness practice.

#### Telephone follow-up

Follow-up telephone calls once a week for 3 weeks by the research assistants will be arranged between the fifth and seventh sessions for participants in the mindfulness group when they have learned the basic mindfulness skills after the four face-to-face sessions to monitor the progress and adherence to home practice of the MBCT participants. To maintain intervention consistency, the research assistants will be reminded not to provide any suggestions or encouragement for the caregivers over the telephone. The telephone follow-up content will be adopted from 3 questionnaires (forms A–C). Form A consists of simple questions such as the frequency of practice and the general learning experience, and form B consists of the same questions as in form A plus some other questions to explore the participants’ application of the practice of mindfulness in daily life. Form C consists of all the questions from forms A and B plus some more questions to explore and monitor the participants’ response to stress after practicing mindfulness and how the participants would apply mindfulness techniques when facing future obstacles.

#### Intervention fidelity

Intervention fidelity checking will be conducted weekly during the intervention period based on the intervention fidelity checklist which covers the rundown and activities of each mindfulness session. The checklist followed the MBCT protocol and was reviewed by an experienced, qualified MBCT teacher. All the intervention sessions will be audio recorded and the investigator will immediately listen to the content after each lesson and check against the fidelity checklist. Achieving a fidelity rate of > 90% will be considered acceptable based on the recommendations of the NIH Behavior Change Consortium [27]. If any missing items in the sessions are found, the interventionist will add it back in the following session. Monthly quality control meetings with all research personnel (the MBCT instructor and research assistants) will be arranged to evaluate their instruction/facilitation skills in this study.

There will be only one interventionist in this study delivering all the modified MBCT sessions and thus variations in the implementation of the interventions will be minimized. The interventionist will be a qualified MBCT teacher who has experience in conducting a similar program to caregivers of PWD. Thus, the interventionist should have a good understanding about the characteristics as well as the difficulties of caregivers for PWD. Since the MBCT protocol has been modified from the original design, the investigators will discuss and clarify with the interventionist about the changes in the protocol and ensure the program is following the outline.

#### Control group (SIRE on dementia care)

The participants of the control group will be provided with chances to socialize and interact with other participants via the SIRE program. The SIRE program has been developed for this study and the aim is to control the socialization and interaction effect as it may mask the effects of the modified MBCT in stress reduction, making it difficult to draw a meaningful conclusion about the effectiveness of the modified MBCT for family caregivers of PWD. The SIRE program consists of seven sessions, with the same group size, duration, and frequency of sessions as the MBCT group, including education sessions on dementia care, caregiver skills training, and group sharing on caregiving tasks. The program will be delivered by an experience nurse in dementia care.

### Data collection procedure

All outcome assessments will be collected by the research assistants, who will be blinded to the group allocation, within 1 week after the completion of the intervention (T1) and 6 months after the intervention (T2). A Data Monitoring Committee will be formed to ensure the safety of the participants and their protection from harm as a result of the intervention. No harmful effect of MBCT was reported in previous studies. The potential harm to the participants may be some negative emotions being triggered when the caregivers share their own personal experience. If this happens, we will refer them to professionals such as social workers or nurses for further assessment. In order to minimize the risks, the interventionist will closely monitor the emotional state of the caregivers. The research assistants and outcomes assessors will also be trained to identify emotional distress of the caregivers and handle emergency situations such as suicide ideation among the caregivers.

### Outcome measurement

A variety of outcomes (described below) will be measured at baseline (T0), immediately after the intervention (T1), and at 6-month follow-up (T2). The FFMQ will be measured in the intervention group only and the baseline measurement will be conducted at the end of the third session (the mid-point of the intervention) when they have learned most of the basic skill and principle of mindfulness [28], followed by measurement immediately after the intervention (T1) and at 6-month follow-up (T2). The previous study showed that the participants’ level of mindfulness started to increase after the mid-point of the intervention, and the remaining sessions after the mid-point will further help the participants to cultivate a mindfulness attitude [29]. All assessments will be carried out by independent outcomes assessors who will be blinded to the group assignments. Sociodemographic data of the subjects will be obtained at baseline (before randomization and within 2 weeks before the implementation of the intervention) to assess the comparability of the groups and thus external validity of the results. Information such as caregiver relationships, time of caring for PWD, employment status, use of caring service, and so on, will be obtained.

### Primary outcome measure

#### Perceived Stress Scale (PSS)

The participants’ perceived stress will be measured with the Chinese version of the PSS [30], which was developed to measure the degree to which situations in one’s life are appraised as stressful [31]. It was used to measure the stress levels in the pilot study. It contains 10 items with a five-point Likert-type scale rating from 0 (never) to 4 (very often). The Chinese version of the PSS was tested in 1800 adults living in the community in Hong Kong [30]. The results showed acceptable levels of psychometric properties which included internal consistency Cronbach’s alpha of 0.85 and a test-retest reliability coefficient 0.85 [30, 32].

### Secondary outcome measures

Previous literature shows that MBCT has a positive effect on several health-related outcomes such as depression, anxiety, and quality of life, and our prior feasibility study also found preliminary effects on the burden and the stress-managing strategies in the family caregivers of PWD [33]. Therefore, the below secondary outcomes were selected.

#### Zarit Burden Scale (ZBI)

Caregiver burden of the participants will be measured with the Chinese version of the ZBI [34]. ZBI was designed to assess the subjective burden of caregivers, defined as the extent to which caregivers perceived their emotional or physical health, social life, and financial status to have changed as a result of caring for their relative with dementia [35]. It comprises 22 items, including factors most frequently mentioned by caregivers as problem areas such as caregivers’ health, psychological well-being, finances, social life, and the relationships between the caregiver and the patient with dementia. It was translated into Chinese in 2005 and test-retest reliability was 0.99 and the split-half correlation coefficient was 0.81 [34]. The correlation between the ZBI and the General Health Questionnaire was 0.59 and 0.57 with the Activity Survey.

#### Center for Epidemiologic Studies Depression Scale (CESDS)

The participants’ depression will be measured with the Chinese version of the CESDS [36], which is a self-reported measure of depression containing 20 items [37]. It measures the common symptoms of depression in terms of depressed mood, feelings of guilt and worthlessness, and feelings of helplessness. The scores range from 0 to 60 and higher CESDS scores indicate increasing levels of depression. The Chinese version of CESD was tested in the community with 3686 Chinese adults attending primary care services [36]. The results showed acceptable levels of psychometric properties which include a test-retest reliability of 0.91 and internal consistency for general depression of 0.86.

#### Hospital Anxiety and Depression Scale-Anxiety (HADS-A)

The level of anxiety will be measured with the Chinese version of the HADS-A [38], which is a seven-item self-report instrument including specific items that assess generalized anxiety including tension, worry, fear, panic, difficulties in relaxing, and restlessness [39]. The scores range from 0 to 21. The Chinese version of HADS-A was tested in 314 Chinese patients with heart disease. The result showed high internal consistency, with Cronbach’s alpha of 0.85 and test-retest reliability of 0.90, respectively [38]. This scale was also validated in the community samples and showed a close correlation with both the Hamilton Rating Scale of Depression and Anxiety (Pearson’s coefficient = 0.67 and 0.63, respectively) [40].

#### Brief Resilience Scale (BRS)

The level of resilience (the ability to bounce back or recover from stress) will be measured by the Chinese version of the BRS [41], which is a six-item, self-report, and five-point rating scale. The scores range from 6 to 30. The Chinese version of the BRS was tested in 349 Chinese undergraduate students. A factor analysis revealed that it contained six factors with a factor loading ranging from 0.68 to 0.91 [42]. The results showed acceptable levels of psychometric properties which included a high internal consistency Cronbach’s alpha of 0.76 [41].

#### Short-form 12-item health survey version 2 (SF-12v2)

The health-related quality of life will be measured by the Chinese version of the SF-12v2 [43], which is a 12-item, self-report, and five-point rating scale. The scores range from 0 to 100. Higher SF-12v2 scores indicate better health-related quality of life. The Chinese version of the SF-12v2 was tested in 2410 Chinese adults who were randomly selected from the general population of Hong Kong. The results showed acceptable levels of psychometric properties which included a high internal consistency Cronbach’s alpha of 0.70 and test-retest reliability of 0.7 [44].

#### Neuropsychiatric Inventory Questionnaire (NPI-Q)

The behavior and psychological symptoms of the care recipient will be measured with the Chinese version of the NPI-Q, which is used to evaluate the frequency, severity, and caregiver distress of 12 neuropsychiatric symptoms including delusions, hallucinations, agitation/aggression, dysphoria/depression, anxiety, euphoria/elation, apathy/indifference, disinhibition, irritability/lability, aberrant motor behaviors, night-time disturbances, and appetite/eating disturbances using a five-point rating scale [45]. The Chinese version of the NPI-Q was tested in 173 patients with cognitive impairment and the results showed acceptable levels of psychometric properties which included a high internal consistency Cronbach’s alpha of 0.76 and test-retest reliability of 0.99 [45].

#### Level of mindfulness

Since the primary objective of the modified MBCT program is to help the participants cultivate a mindfulness attitude, the level of mindfulness will be measured as the process indicator using the Chinese version of the Five Facets Mindfulness Questionnaire Short Form (FFMQ-SF) [46], which has been commonly used to measure mindful awareness in mindfulness studies [47]. It is a self-report questionnaire measuring the five facets of mindfulness which includes observing, describing, acting with awareness, nonjudging of and nonreactivity to inner experience (e.g., “I’m good at finding words to describe my feelings”). The higher the score, the higher level of mindfulness. The Chinese version of the FFMQ-SF was tested in community-dwelling Chinese adults (*n* = 230) and clinical patients with significant psychological distress (*n* = 156) [46]. The results showed good test-retest reliability of 0.82 and a high internal consistency Cronbach’s alpha 0.80 in the community and clinical samples [46].

The adherence rate and frequency of mindfulness practice will be assessed based on the participants’ attendance in the weekly mindfulness training sessions in the experimental group. Additionally, they will be instructed to record the frequency and duration of mindfulness practice at home during the previous week. They will also be asked to write down weekly mindfulness practice duration (hours) and rate their overall level of adherence to their practice regimen on a 10-point scale from 0 (non-compliance) to 10 (full compliance) based on the following question: ‘Did I comply with the practice regimen in the past 7 days?’. This information will be collected weekly after the mindfulness program and will continue to be collected bi-weekly during the follow-up period. The reasons for any drop-out of each participant will also be recorded.

### Data analysis plan

IBM SPSS 23.0 software will be used for the data analysis. The Kolmogorov–Simonov test will be used for checking normality. To ensure the similarity of the subjects in the two groups (modified MBCT and SIRE) at baseline, the demographics, adherence rate, frequency of mindfulness practice, and the outcome measurements will be compared by using a *t* test for continuous variables and a Chi-square test for categorical variables. Covariates will be controlled in the main analyses if a significant difference of variables is found at baseline. Both per-protocol (PP) and intention-to-treat (ITT) analyses will be performed. In the PP population, we will include the subjects who attend > 70% of the intervention sessions. A sensitivity test will be performed to compare the differences between the ITT and PP results. The aim of conducting both ITT and PP analyses is to uncover factors affecting the use and effectiveness of the modified MBCT, such as noncompliance and acceptability [48].

Descriptive statistic will be used to analyze the recruitment rate, attrition rate, duration, patterns, and consistency of the participants practicing mindfulness to indicate the acceptability of the MBCT. Mixed multivariate modeling (MANOVA) tests will be adopted to investigate the between-group effect (groups: modified MBCT and SIRE), the within-group effect (times: T0, T1, and T2), and the interaction time effect of group by time effect (group × time) on all of the outcome variables including PSS, HADS-A, CESD, ZBI, BRS, NPI-Q, and SF-12v2, and the process indicator (FFMQ). Correlation checking by Pearson’s product moment correlation of all the psychological outcomes including PSS, HADS-A, CESD, ZBI, BRS, NPI-Q, SF-12v2, and the process indicator (FFMQ) at baseline will be performed. If the correlation is too strong (Pearson’s *r* > 0.6), which may result in multicollinearity and violation of the assumption of mixed repeated MANOVA, then two-way repeated ANOVA and Bonferroni’s adjustments will be adopted to prevent a type I error [49]. Post-hoc pairwise comparisons using Tukey’s Honestly Significant Difference (HSD) will be performed to examine the significant difference in the outcomes.

The Pearson’s correlation coefficient (*r*) (or Spearman’s correlation coefficient, as appropriate) will be adopted for the following variables to examine their correlations:

1. The FFMQ total score as the independent variable and the outcome measurements including mean scores of stress, depression, burden, and anxiety as the dependent variables.
2. The subscale (each facet) of the FFMQ score as the independent variable and the outcome measurements including mean scores of stress, depression, burden, and anxiety as the dependent variables.
3. The duration of mindfulness practice as the independent variable and the level of mindfulness as the dependent variable.

The degree and pattern of missing data will be analyzed using SPSS Statistics Missing Values. If the missing value is a small amount (< 20%) and randomly distributed, the last observation carried forward (LOCF) method will be adopted to replace the missing data [50, 51]. However, if the missing values are not randomly distributed, multiple imputations will be adopted. This will create several different plausible imputed datasets and the results obtained from each of the sets will be appropriately combined [52].

## Discussion

The number of PWD worldwide is projected to triple to 131.5 million by 2050 [53]. As the disease progresses, the self-care ability of PWD will gradually reduce or be lost. The family caregivers of PWD face many challenges since they need to balance the caregiving tasks with other demands, such as their career and social life. The continuous stress of caregiving results in different physical and psychological comorbidities among these caregivers. In a previous study, the stress-related symptoms including depression and anxiety were found in 45% to 85% of caregivers of PWD, respectively [54, 55]. Improving the psychological well-being of family caregivers is becoming important in dementia care.

MBCT aims to help caregivers develop accepting and nonjudgmental attitudes to caregiving, resulting in stress reduction and also promoting the well-being of the family caregivers. The MBCT does not emphasize any changes in personal thoughts or feelings, but assists and teaches the caregivers to accept their negative emotions and keep them at a distance. This unique nature of mindfulness may empower the caregivers who are facing everyday stress. The improvement in the mental health of the family caregivers could maintain their sustainability in providing daily care to PWD for a longer period of time, delay the institutionalization of PWD, and also decrease the cost of care to society.

The improvement in the mental health of the family caregivers may also extend to PWD. The mindfulness practice can help the caregiver facilitate their responses to external stimuli, such as the behavioral problems of PWD, in a less reactive and impulsive way. In our previous feasibility study, a number of caregivers found that their acceptance of the behavioral problems (e.g., wondering, asking repeated questions) of the PWD was increased after practicing mindfulness and they showed more patience in managing the caring problem [16]. Being more mindful, an example caregiver shared that she could avoid arguing with the PWD but be silent for a while when her mother asked her questions repeatedly, thus resulting in a more harmonious relationship with the PWD and less behavioral problems for the PWD. The interaction of the caregivers and PWD could affect the severity of the BPSD [56]. Since only a few previous studies have explored the use of mindfulness in the caregivers, this is the first study that will also look at the impact on the BPSD of the PWD.

## Limitations

We are conducting this study in nongovernmental organizations offering support for the family caregivers, which may limit the ability to generalize the findings to all community-dwelling caregivers. There is also a chance of bias since the participants are aware of the intervention. Thus, the level of mindfulness will be measured to understand the mediating effect or active components of the MBCT contributing to any significant benefits for the participants.

## Conclusion

According to the recent systematic review, only a few clinical trials have reported a preliminary and short effect of mindfulness in the family caregivers of PWD. To meet the needs for the family caregivers of PWD, this proposed study has collected feedback from family caregivers of PWD and addressed the limitations (i.e., a low adherence rate) from previous studies by modifying the original MBCT protocol. The modified MBCT protocol aims to facilitate and support the family caregivers by cultivating a mindful attitude, empowering themselves to manage their stress, and promoting their resilience. The stress of family caregivers can last a long time, and hence examining the sustainable effects of the modified MBCT (i.e., a 6-month follow-up) is important. This study will also collect other important information on the intervention, including the participants’ adherence to mindfulness practices, level of mindfulness, and qualitative data for better understanding the active ingredients of the modified MBCT. The findings from this proposed randomized controlled trial could provide scientific evidence for the effectiveness of the modified MBCT for family caregivers of PWD. If significant benefits are found, the modified MBCT can be used as a standard supportive intervention for the family caregivers of PWD.

## Trial status

Recruitment commenced in May 2018, and the trial is scheduled to end in May 2019.

## Additional file

Additional file 1: SPIRIT 2013 checklist: recommended items to address in a clinical trial protocol and related documents. (DOC 124 kb)

## Abbreviations

BPSD: Behavioral and psychological symptoms of dementia
BRS: Brief Resilience Scale
CESDS: Center for Epidemiologic Studies Depression Scale
FFMQ: Five Facet Mindfulness Questionnaire
HADS-A: Hospital Anxiety and Depression Scale-Anxiety
ITT: Intention-to-treat
MBCT: Mindfulness-based cognitive therapy
MBI: Mindfulness-based intervention
MBSR: Mindfulness-Based Stress Reduction
NPI-Q: Neuropsychiatric Inventory Questionnaire
PP: Per-protocol
PSS: Perceived Stress Scale
PWD: People with dementia
SF-12v2: Short-form 12-item health survey version 2
SIRE: Social interaction and routine education
ZBI: Zarit Burden Scale
